# VSTM1 regulates monocyte/macrophage function via the NF-κB signaling pathway

**DOI:** 10.1515/med-2021-0353

**Published:** 2021-10-11

**Authors:** Xiao-Fei Wang, En-Zhou, Dong-Jiu Li, Cheng-Yu Mao, Qing He, Jun-Feng Zhang, Yu-Qi Fan, Chang-Qian Wang

**Affiliations:** Department of Cardiology, The Ninth People’s Hospital, Shanghai Jiao Tong University School of Medicine, Bao Shan, Shanghai 201900, People’s Republic of China; Department of Cardiology, The Ninth People’s Hospital, Shanghai Jiao Tong University School of Medicine, 280 Mo He Road, Bao Shan, Shanghai 201900, People’s Republic of China

**Keywords:** VSTM1, monocyte/macrophage, inflammation, atherosclerosis, NF-κB

## Abstract

**Objective:**

V-set and transmembrane domain-containing protein 1 (VSTM1) is negatively correlated with inflammation. However, its effect on atherosclerosis (AS) remains largely unexplored. In this study, we aimed to assess the effect of VSTM1 on the biological function of human peripheral blood mononuclear cells /macrophages stimulated by oxidized low-density lipoprotein (ox-LDL).

**Methods:**

U937 cells were divided into three groups as follows: control group, pLenti-VSTM1 shRNA group (VSTM1 depletion), and pLenti-VSTM1 group (VSTM1 overexpression). Cellular migration, chemotaxis, apoptosis, and secretion of inflammatory factors of monocytes/macrophages stimulated by ox-LDL were studied.

**Results:**

Overexpression of VSTM1 decreased the proliferation of U937 cells and induced cellular apoptosis. Depletion of VSTM1 enhanced the invasiveness and chemotaxis, increased the inflammatory response, and reduced the incidence of cell necrosis and apoptosis. Nuclear factor κ of B cells (NF-κB) was activated in VSTM1-depleted U937 cells.

**Conclusion:**

VSTM1 might play an important role in the activation of monocytes/macrophages and participate in the pathogenesis of AS via regulating NF-κB activity.

## 1 Introduction

As one of the most common cardiovascular diseases in the world, coronary heart disease (CHD) can cause myocardial infarction and seriously endanger human health. Atherosclerosis (AS) is considered as the pathological basis of CHD. It has been found that monocyte/macrophage activity is the key factor contributing to the pathogenesis of AS [1,2,3]. Monocytes play an important role in AS. It has been described that monocytes are accumulated into vascular endothelial cells and turn into macrophages through the phagocytosis of oxidized low-density lipoprotein (ox-LDL) [4].

Earlier studies have shown that the activation of biological functions of monocytes and macrophages and the cascade reaction of inflammation in AS are regulated by a complex network system, including inflammation and apoptosis signaling pathway, which requires the activation of multiple transcription factors, such as activator protein 1, nuclear factor κ of B cells (NF-κB), signal transducer and activator of transcription 1/3, nuclear factor of activated T cells, hypoxia-inducible factor 1, p53, and so on [5]. As a result of the complexity of network regulation, the functional regulation of monocytes is still unclear up to now. Therefore, it is necessary to explore the main target regulating the activation of monocytes/macrophages. Genes that can simultaneously regulate inflammation, apoptosis, lipid phagocytosis, migration, and invasion are still a research hotspot in the prevention and treatment of acute cardiovascular events.

V-set and transmembrane domain-containing protein 1 (VSTM1), also known as signal inhibitory receptor on leukocytes-1, is a type of membrane receptor [6,7] located on the human chromosome 19q.13.4, which is adjacent to the leukocyte receptor complex. VSTM1 is highly similar to many receptor proteins, such as leukocyte-associated immunoglobulin-like receptor 1 which regulates the function of leukocytes [8]. As a membrane receptor, VSTM1 consists of extracellular, transmembrane, and intracellular sequences. Its intracellular sequence is highly conserved in different species and genera, indicating that such sequence is important for the function of VSTM1 [9,10]. Previous research has found that VSTM1 is negatively correlated with inflammation [11]. In peripheral blood mononuclear cells (PBMCs), the expression of tumor necrosis factor-α (TNF-α) in monocytes with high expression of VSTM1 is significantly lower than that in monocytes with low expression of VSTM1 [12]. In patients with pneumonia, the individuals with high expression of VSTM1 in neutrophils suffer more serious clinical symptoms than those with low expression of VSTM1 [8].

The role of VSTM1 in cardiovascular disease remains largely unexplored. In this study, we aimed to investigate the regulatory role of VSTM1 in monocytes, clarify the potential mechanism involved, and reveal its effect on the pathogenesis of AS.

## 2 Materials and methods

### 2.1 Construction of U937 stable mutants with VSTM1 overexpression/depletion

#### 2.1.1 Culture of U937 cells and cell transfection

The U937 cell was purchased from Yuanchuang Biotechnology Co., Ltd (Shanghai, China). Cells were maintained in Gibco Roswell Park Memorial Institute-1640 medium (Rochester, NY, USA) supplemented with 10% fetal bovine serum, streptomycin (100 µg/mL), and penicillin (100 U/mL) at 37°C in a humidified atmosphere containing 5% CO_2_. The cells were subcultured when a confluence of 90% was achieved. After two to four generations, cells were used for further experiments.

#### 2.1.2 Cell transfection

Cells were seeded into a 12-well plate and transfected with VSTM1 siRNA (100 nM) or plasmid DNA (100 µM), using Lipofectamine 3000 transfection reagent (Invitrogen, Cat. No. 11668019, ThermoFisher Scientific, Waltham, MA, USA) following the manufacturer’s instructions. Experiments were performed 24 h after the transfection.

#### 2.1.3 Depletion of VSTM1

siRNA targeting VSTM1 was used to silence the endogenous expression of VSTM1. siRNA was purchased from Zorin Shanghai Co., Ltd. (Cat. No. ZR-1-000130, Shanghai, China). VSTM1 shRNA was designed as follows: top strand 5′- CACCGGTTGAAGCCGAGAGCAATGTCGAAACATTGCTCTCGGCTTCAACC -3′; bottom strand 5′-AAAAGGTTGAAGCCGAGAGCAATGTTTCGACATTGCTCTCGGCTTCAACC-3′. Briefly, 5.5 μg VSTM1 shRNA/VSTM1 plasmid, 7.5 μg psPAX2 packaging plasmid, 2 μg envelope plasmid pMD2G, 45 μL Polyjet, and 100 µL serum-free Eagle s minimum essential medium were combined to construct virus. siRNA (100 µM) and its negative control (100 µM) were transfected into U937 cells using Lipofectamine 3000.

#### 2.1.4 Efficiency of plasmid transient transfection assay

Overexpression plasmids were constructed by our laboratory using the pBABE vector. The interference plasmid was purchased from Shanghai Jikai Company. The constructed plasmid was transiently transfected for 24 h, and polyethylenimine was used as the transfection reagent.

#### 2.1.5 Construction of stable transgenic plants

Retrovirus was used for gene overexpression, and lentivirus was used for gene depletion. Infected cells (U937) were packaged with the virus, and the efficiency of amplified samples was determined after stable strains were obtained.

### 2.2 Viral infection

Various viral plasmids were transfected into 293 T cells, and virus packaging vectors (MD2G, PSPAX2) were used for virus packaging. Viral supernatants were collected after 48–72 h, centrifuged and concentrated, and multiplicity of infection (MOI) was determined by gradient dilution method. Monocytes/macrophages were infected at an MOI of 10.

The lentiviral vectors pLenti-VSTM1 (VSTM1 overexpression; its skeleton name was PLVX) and pLenti-VSTM1 shRNA (VSTM1 depletion; its skeleton name was Pll3.7) were used to infect monocytes/macrophages, followed by stimulation by ox-LDL (100 μg/mL) for 4 h according to the manufacturer’s instructions. Meanwhile, control groups (noninfected monocyte/macrophage group, noninfected ox-LDL stimulation group, PLVX or Pll3.7 empty plasmid vector group, and PLVX or Pll3.7 empty plasmid vector ox-LDL stimulation group) were set up to validate the overexpression and depletion of VSTM1. The effects of ox-LDL-exposed monocytes/macrophages on the migration, invasion, apoptosis, secretion of inflammatory factors, reactive oxygen species (ROS), and other oxygen-free radicals were studied.

### 2.3 Experimental group

The experimental groups were divided as follows: Con group was referred to U937 monocyte without any experimental intervention; NC group was referred to U937 monocyte infected with pLenti-VSTM1 negative control sequence; and Sh-VSTM1 group was referred to U937 monocyte infected with pLenti-VSTM1 shRNA.

### 2.4 Enzyme-linked immunosorbent assay (ELISA)

The levels of interleukins (ILs), interferon-α (IFN-α), TNF-α, transforming growth factor-β, high mobility group box 1 (HMGB1), and matrix metalloproteinase-9 (MMP-9) in cells were determined by respective ELISA kits (ThermoFisher Scientific, Waltham, MA, USA) according to the manufacturer’s instructions. Briefly, samples and standards were added into a microplate precoated with a monoclonal antibody. After the removal of enzyme reagent and any unbound antibody, a substrate solution was added to each well, a stop solution was used to terminate color development, and the color intensity in each well was determined at a wavelength of 450 nm.

### 2.5 Chemotaxis assay and invasion test

#### 2.5.1 Chemotaxis assay

Briefly, 1 × 10^5^ cells were seeded onto the top side of 8 µm pore polycarbonate membrane in a 24-well transwell unit, serum-free medium containing chemokines membrane cofactor protein-1, platelet-derived growth factor-BB (PDGF-BB), or vascular endothelial growth factor was placed into the bottom chamber, and the cells were incubated at 37°C for 3 h. Subsequently, the membrane was removed and stained with hematoxylin–eosin (H&E), and the number of stained cells outside the membrane was determined under a microscope. The changes in invasive chemotactic ability were assessed.

#### 2.5.2 Invasion test

Briefly, 1 × 10^5^ cells were seeded onto the top side of a 24-well transwell unit, which was coated with 5 μg/mL matrix gel. Serum-free medium was placed in the bottom chamber, and the transwell unit was incubated at 37°C in an incubator containing 5% CO_2_. After 24 h, the membrane was subjected to H&E staining. Cells on the membrane were observed under a microscope and compared with the control group. The changes in invasive ability were assessed.

### 2.6 Apoptosis and ROS production

2,7-Dichlorodihydrofluorescein diacetate (DCFH-DA) probe kit (ThermoFisher Scientific, Waltham, MA, USA) was used to detect the ROS level in monocytes, and flow cytometry was used to detect the apoptotic rate of Annexin V/Propidium Iodide double-stained monocytes/macrophages.

### 2.7 Effects of VSTM1 and Src homology region 2 domain-containing phosphatase 1 on the proliferation of monocytes/macrophages stimulated by ox-LDL: cell counting kit-8 (CCK-8) assay

Cells were seeded into a 96-well plate at a density of 1,000–5,000 cells/well. The marginal pore was filled with sterile PBS, and cells were cultured at 37°C with 5% CO_2_. After 24 h, 10 μL CCK-8 solution was added into each well, followed by incubation at 37°C for 1 h. The absorbance at a wavelength of 450 nm was measured by enzyme labeling.

### 2.8 Western blotting analysis

Total proteins were extracted from U937, U937 NC, and U937 cells overexpressing VSTM1 using RIPA lysis buffer. Equal amounts of proteins were subjected to sodium dodecyl sulfate–polyacrylamide gel electrophoresis using 7.5% gels and electrotransferred onto polyvinylidene fluoride membranes. The blots were incubated with primary antibodies against VSTM1 (1:1,000, H00284415-M03, Abnova), p-p65/NF-κB (1:1,000, ab86299, Abcam), and p65/NF-κB (1:1,000, ab32536, Abcam), followed by incubation with appropriate horseradish peroxidase-conjugated secondary antibodies. Immunoreactive bands were visualized using the efficient chemiluminescence reagent kit (Millipore Corp., United States). The blot intensities were quantified with Image J software (Image J, RRID: SCR_003070).

### 2.9 Data analysis

The quantitative data were expressed as mean ± standard error. The results were analyzed by analysis of variance (ANOVA), followed by the least significant difference test for post hoc comparison using the SPSS 13.0 software (SPSS, RRID: SCR_002865). A *p* value < 0.05 was considered statistically significant.

## 3 Results

### 3.1 Construction of U937 stable strains with VSTM1 overexpression/depletion

#### 3.1.1 Plasmid expression screening

**Figure 1:**
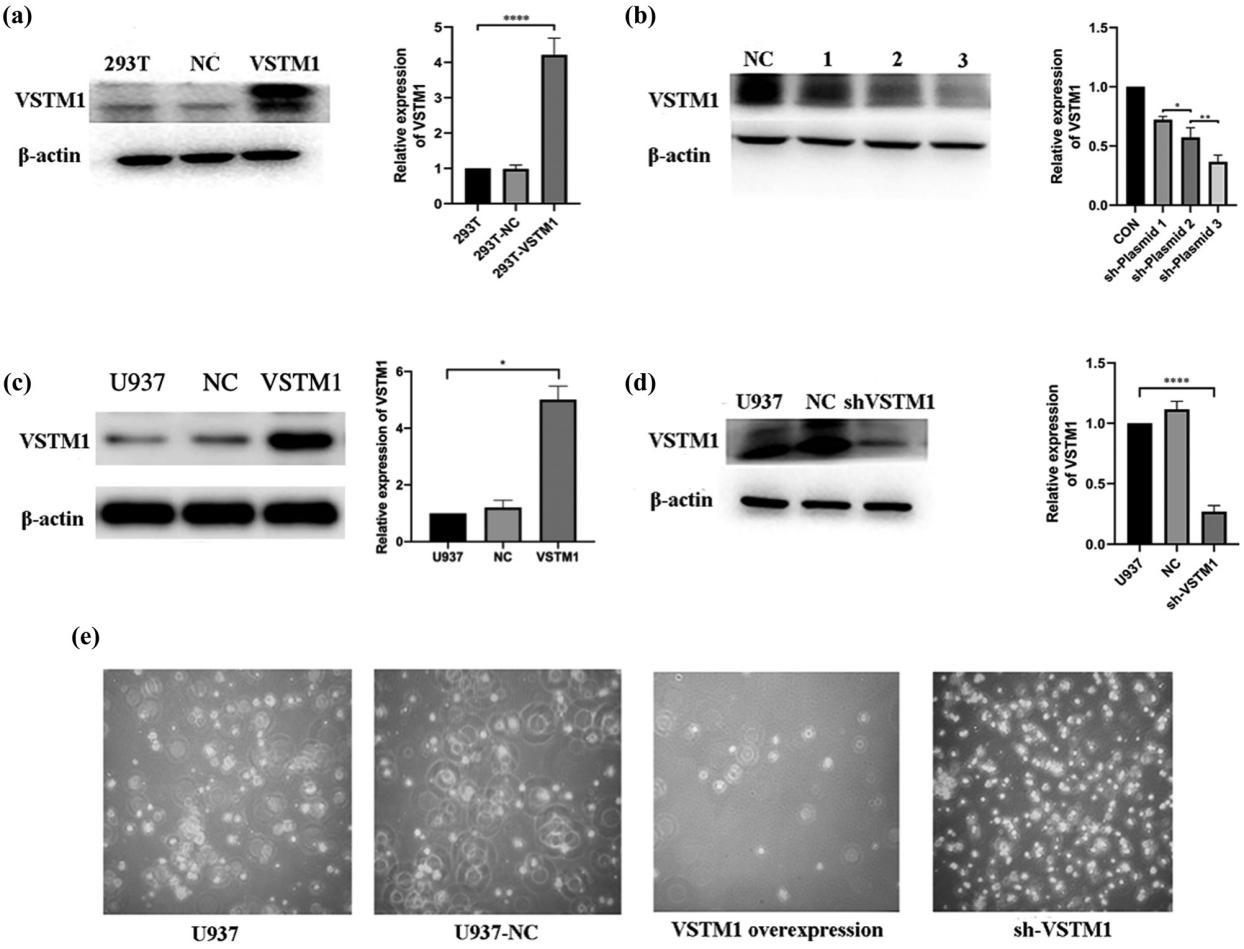
Plasmid construction and identification of transfection efficiency. (a) VSTM1 overexpression plasmid was transfected into 293 T cells successfully (NC as control plasmid), and the expression of VSTM1 at the protein level was evaluated by Western blotting analysis (compared with the 293 T group, *p < 0.05, **p < 0.01, ***p < 0.001, ****p < 0.0001, *n* = 3). (b) Three types of VSTM1 interference plasmids were transfected into 293 T cells. The transfection efficiency was compared between them (Con as control plasmid, 1, 2, 3 as plasmid number, compared with the plasmid 2 group, *p < 0.05, **p < 0.01, ***p < 0.001, ****p < 0.0001, *n* = 3). (c) Evaluation of transfection efficiency of VSTM1 overexpression stable cell lines (NC as control plasmid), and the expression of VSTM1 at the protein level was evaluated by Western blotting analysis (compared with the 293 T group, *p < 0.05, **p < 0.01, ***p < 0.001, ****p < 0.0001, *n* = 3). (d) Evaluation of transfection efficiency of VSTM1 interference stable cell lines (NC as control plasmid), and the expression of VSTM1 at the protein level was evaluated by Western blotting analysis (compared with the 293 T group, *p < 0.05, **p < 0.01, ***p < 0.001, ****p < 0.0001, *n* = 3). (e) VSTM1 overexpression stable strains grew very slowly. Therefore, only the interference group was used in the subsequent experiment. Statistical analysis was performed using Pearson chi-square test or Fisher’s exact test (*n* < 5) with subsequent multiple comparisons using chi-square with Bonferroni correction for categorical variables. One-way ANOVA with subsequent post hoc multiple comparisons was used for continuous variables with the Student–Newman–Keuls test.

The expression of VSTM1 was significantly increased after the VSTM1 overexpression plasmid was transfected into 293 T cells (1.00 vs 4.23 ± 0.54; compared with the 293 T group, *n* = 6, *p* ＜ 0.0001; Figure 1a). The gene silencing efficiency of plasmid 3 was the most significant (0.63 ± 0.19 vs 0.42 ± 0.06; compared with sh-plasmid 2, *n* = 6, *p* = 0.0052; Figure 1b). Therefore, plasmid 3 was selected for further experiments.

#### 3.1.2 Construction of stable transgenic strains

Compared with U937 cells, the expression of VSTM1 in U937 cells overexpressing VSTM1 was significantly increased (compared with the U937 group, *n* = 6, *p* ＜ 0.0001; Figure 1c). The expression of VSTM1 in the interference group was significantly lower compared with the control group (1.00 vs 0.24 ± 0.03; compared with the U937 group, *n* = 6, *p* ＜ 0.0001; Figure 1d), indicating that the transfection efficiency of the interference group was high. After VSTM1 was overexpressed in U937 cells (sh-VSTM1 group), the growth rate of cells was significantly decreased (compared with the U937 group, *n* = 6, *p* < 0.0001; Figure 1e), accompanied by increasing numbers of apoptotic and necrotic cells. The interference group was used in further experiments.

### 3.2 The effects of VSTM1 on the function of monocytes/macrophages stimulated by ox-LDL

#### 3.2.1 Invasiveness and chemotaxis of monocytes/macrophages

##### 3.2.1.1 Invasion test results of VSTM1 overexpression group and VSTM1 depletion group

Due to the relatively small number of cells and a large number of apoptotic and necrotic cells after ox-LDL stimulation, it was impossible to get enough cells for the transwell test. Therefore, only the invasive and chemotactic changes of U937 cells without ox-LDL treatment were assessed.

**Figure 2:**
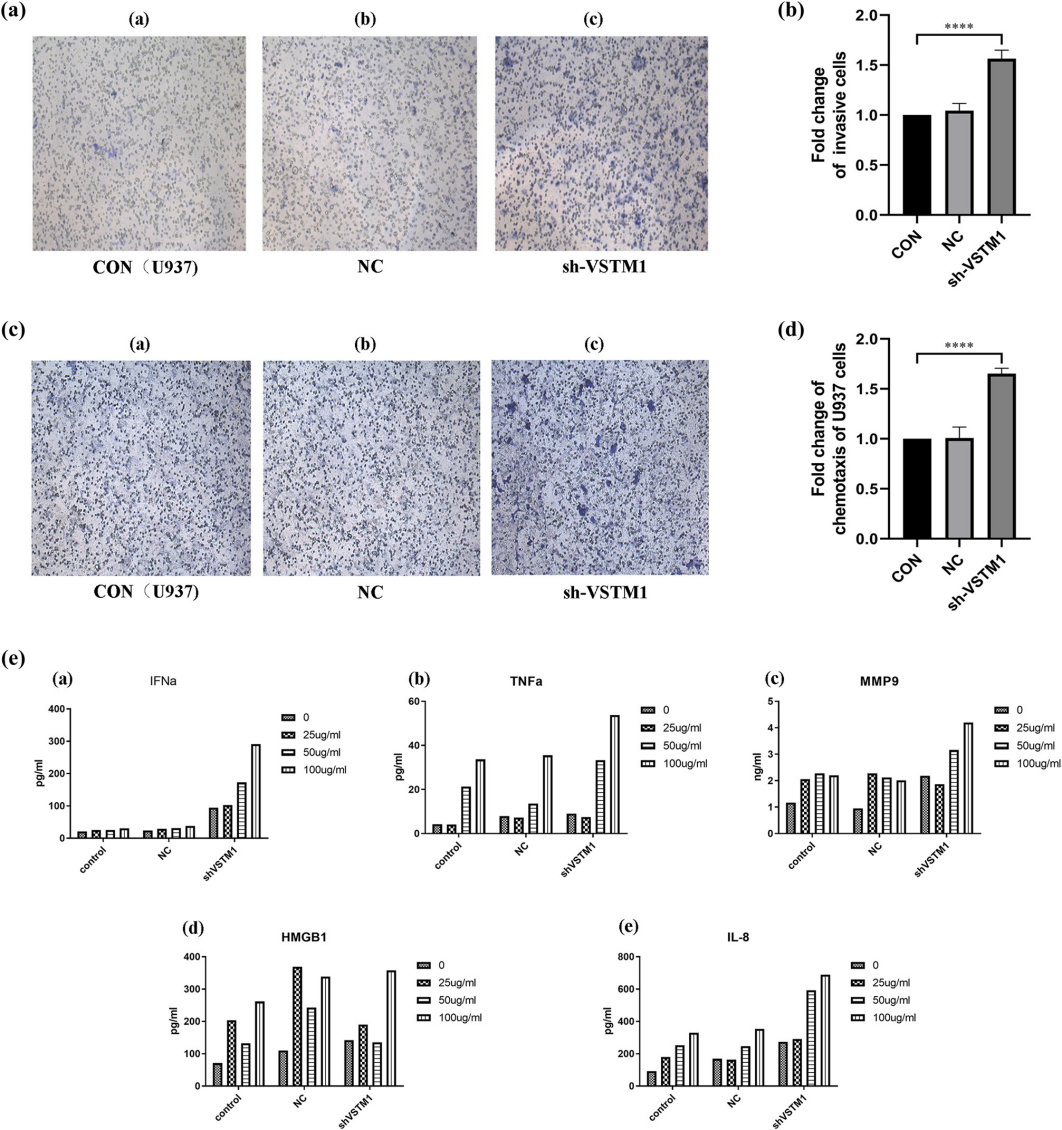
Effects of sh-VSTM1 on invasiveness, chemotactic ability, and cellular inflammatory response. (a and b) Invasion results of U937 , U937 NC, and sh-VSTM1 U937 cells without exposure to ox-LDL (compared with the 293 T group, *p < 0.05, **p < 0.01, ***p < 0.001, ****p < 0.0001, *n* = 5). (c and d) Chemotaxis results of U937, U937 NC, and sh-VSTM1 U937 cells without exposure to ox-LDL (compared with the 293 T group, *p < 0.05, **p < 0.01, ***p < 0.001, ****p < 0.0001, *n* = 3). (e) IFN-α, TNF-α, MMP-9, HMGB1, and IL-8 concentrations in cell supernatant. Statistical analysis was performed using Pearson chi-square test or Fisher’s exact test (*n* < 5) with subsequent multiple comparisons using chi-square with Bonferroni correction for categorical variables. One-way ANOVA with subsequent post hoc multiple comparisons was used for continuous variables with the Student–Newman–Keuls test.

The invasiveness of U937 cells in the sh-VSTM1 group was significantly enhanced compared with U937 cells and U937 NC cells, suggesting that depletion of VSTM1 could significantly enhance the invasiveness of U937 cells (1.00 vs 1.51 ± 0.13; compared with the control group, *n* = 6, *p* ＜ 0.0001; Figure 2a and b).

##### 3.2.1.2 Chemotaxis test results of VSTM1 depletion group stimulated by ox-LDL (PDGF-BB as chemokine)

The chemotaxis of U937 cells in the sh-VSTM1 group was significantly enhanced compared with U937 cells and U937 NC cells, suggesting that depletion of VSTM1 could significantly increase the chemotaxis of U937 cells (1.00 vs 1.63 ± 0.14; compared with the control group, *n* = 6, *p* ＜ 0.0001; Figure 2c and d).

#### 3.2.2 Effects of VSTM1 on the production of inflammatory chemokines in U937 cells

The concentration of IFN-α in the supernatant of U937 cells and U937 NC cells was relatively low (about 20 pg/mL), and there was no significant increase after treatment with ox-LDL. In the sh-VSTM1 group, the concentration of IFN-α was significantly higher (about 100, 150, and 300 pg/mL) compared with U937 cells and U937 NC cells after treatment with ox-LDL (25, 50, and 100 μg/mL; Figure 2e(a)).

The concentration of TNF-α in the supernatant of U937 cells (about 5 pg/mL) and U937 NC cells (7 pg/mL) was relatively low, which was significantly increased (35 pg/mL in U937 cells and 38 pg/mL in U937 NC cells) after treatment with ox-LDL (100 μg/mL) in a dose-dependent effect. In the sh-VSTM1 group, the concentration of TNF-α in the supernatants was about 55 pg/mL after treatment with ox-LDL (100 μg/mL; Figure 2e(b)).

The concentration of MMP-9 in U937 cells and U937 NC cells was slightly increased (about 2 ng/mL) after treatment with ox-LDL, and the concentration of MMP-9 in the sh-VSTM1 group was higher (about 3 and 4 ng/mL) after treatment with ox-LDL (50 and 100 μg/mL; Figure 2e(c)).

Figure 2e(d) shows that the concentration of HMGB1 in the supernatant was slightly increased in each group after treatment with ox-LDL.

Figure 2e(e) reveals that the concentration of IL-8 in the supernatant of U937 cells (100 pg/mL) and U937 NC cells (150 pg/mL) was low. After the exposure to ox-LDL, the concentration of IL-8 was slightly increased in a dose-dependent manner. In the sh-VSTM1 group without ox-LDL exposure, the concentration of IL-8 was about 300 pg/mL, which was higher compared with U937 cells and U937 NC cells. After treatment with ox-LDL (100 μg/mL), the concentration of IL-8 was significantly increased (about 650 pg/mL).

#### 3.2.3 Effect of VSTM1 on ROS production in U937 cells

**Figure 3:**
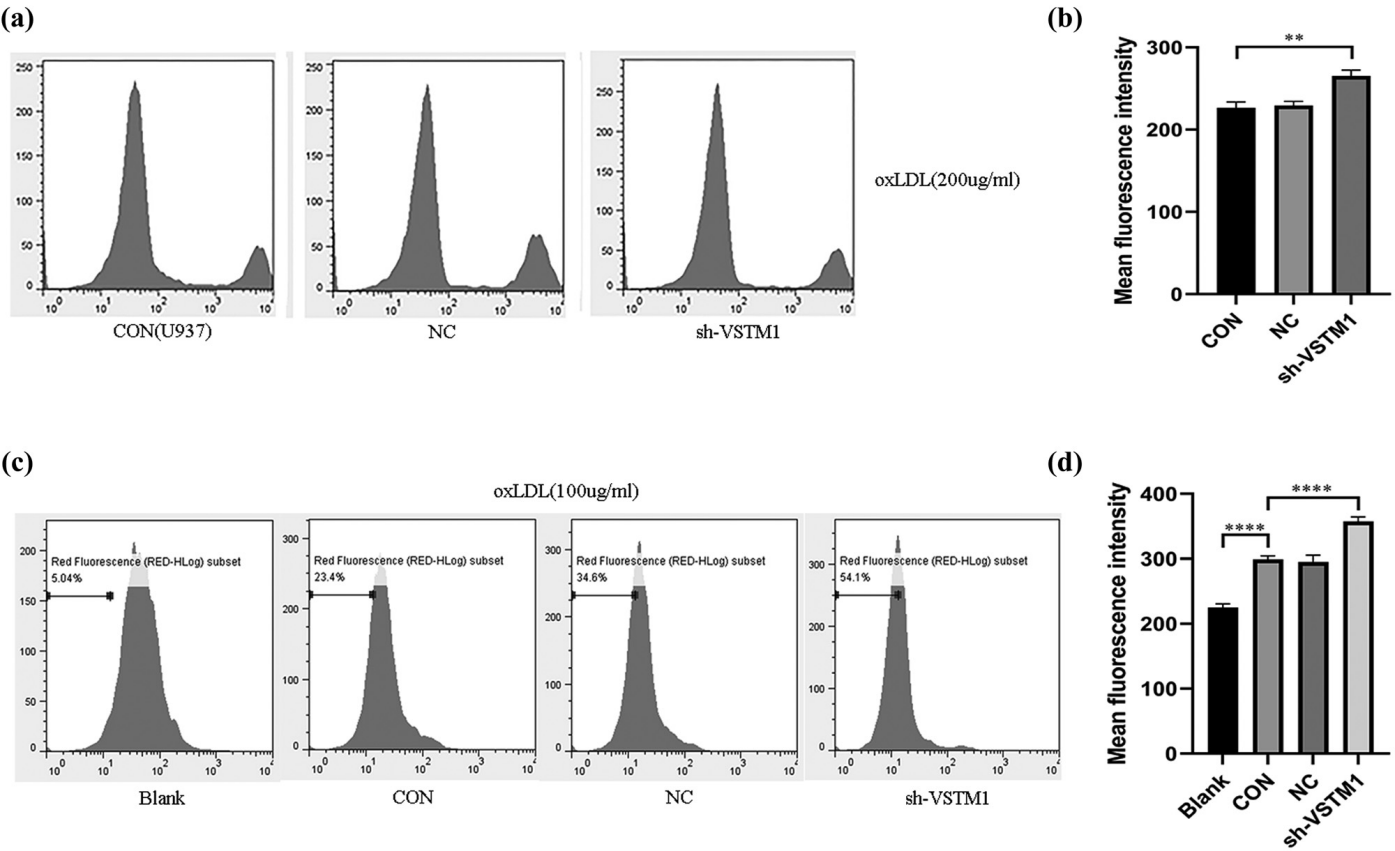
Effects of sh-VSTM1 on ROS production and lipid phagocytosis of monocytes/macrophages stimulated by ox-LDL. (a) and (b) Effects of ox-LDL on ROS production in U937, U937 NC, and sh-VSTM1 U937 cells (compared with the 293 T group, *p < 0.05, **p < 0.01, ***p < 0.001, ****p < 0.0001, *n* = 5). (c) and (d) Effect of VSTM1 on lipid phagocytosis of monocytes/macrophages stimulated by ox-LDL in U937, U937 NC, and sh-VSTM1 U937 cells (compared with the blank group and control group, *p < 0.05, **p < 0.01, ***p < 0.001, ****p < 0.0001, *n* = 5). Statistical analysis was performed using Pearson chi-square test (*n* ≥ 5) or Fisher’s exact test (*n* ＜ 5) with subsequent multiple comparisons using chi-square with Bonferroni correction for categorical variables. One-way ANOVA with subsequent post hoc multiple comparisons was used for continuous variables with the Student–Newman–Keuls test.

The production of ROS in the sh-VTSM1 group was significantly increased (266.42 ± 13.45) compared with U937 cells (219.45 ± 11.89) and U937 NC cells (220.13 ± 10.76; *n* = 6, *p* = 0.0012; Figure 3a and b).

#### 3.2.4 Effect of VSTM1 on lipid phagocytosis of monocytes/macrophages

The fluorescence intensity of the control group and NC group was comparable (295.13 ± 5.61 vs 293.29 ± 6.31), which was higher compared with the cells without ox-LDL exposure (blank group; 213.56 ± 4.87; *n* = 6, *p* ＜ 0.0001). However, the fluorescence intensity of the sh-VSTM1 group was significantly increased (342.84 ± 4.57) compared with the control group (*n* = 6, *p* ＜ 0.0001). These results indicated that depletion of VSTM1 enhanced the capacity of lipid phagocytosis of U937 cells (Figure 3c and d).

### 3.3 Effects of VSTM1 on apoptosis and proliferation of U937 cells

#### 3.3.1 Apoptosis and necrosis of U937 cells

**Figure 4:**
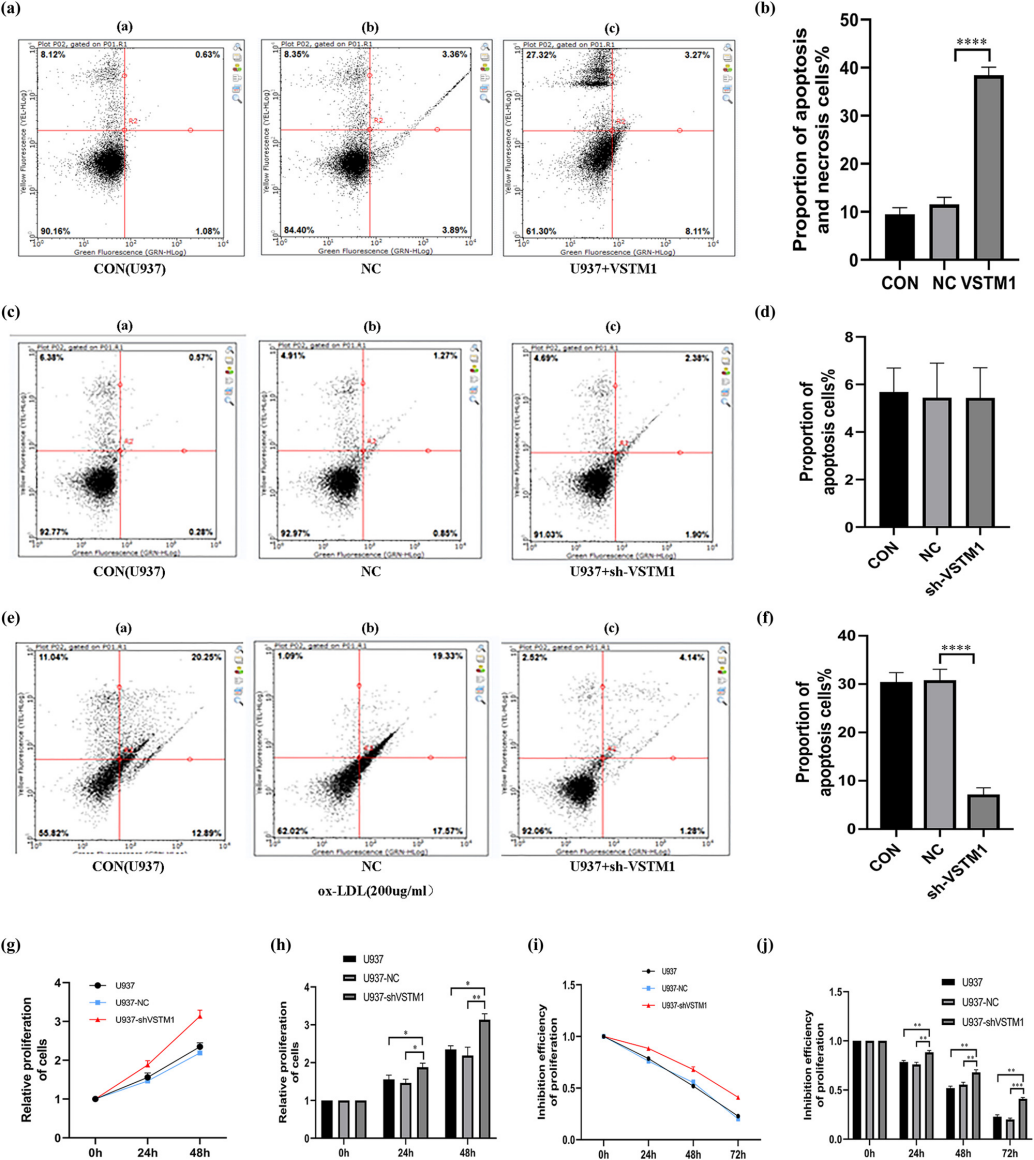
Effects of VSTM1 on apoptosis and proliferation of monocytes/macrophages stimulated by ox-LDL. (a and b) Detection of apoptosis after VSTM1 was overexpressed in U937 cells (compared with the NC group, *p < 0.05, **p < 0.01, ***p < 0.001, ****p < 0.0001, *n* = 3). (c and d) Apoptosis of U937, U937 NC, and sh-VSTM1 U937 cells without exposure to ox-LDL (compared with the control group, *p < 0.05, **p < 0.01, ***p < 0.001, ****p < 0.0001, *n* = 3). (e and f) Apoptosis of U937, U937 NC, and sh-VSTM1 U937 cells exposed to ox-LDL (compared with the NC group, *p < 0.05, **p < 0.01, ***p < 0.001, ****p < 0.0001, *n* = 5). (g) and (h) Proliferation of U937, U937 NC, and sh-VSTM1 U937 cells without exposure to ox-LDL (compared with the control group, *p < 0.05, **p < 0.01, ***p < 0.001, ****p < 0.0001, *n* = 5 and the NC group, *p < 0.05, **p < 0.01, ***p < 0.001, ****p < 0.0001, *n* = 5). (i) and (j) Inhibitory effect of ox-LDL on the growth of U937, U937 NC, and sh-VSTM1 U937 cells (compared with the control group, *p < 0.05, **p < 0.01, ***p < 0.001, ****p < 0.0001, *n* = 5 and compared with the NC group, *p < 0.05, **p < 0.01, ***p < 0.001, ****p < 0.0001, *n* = 5). Statistical analysis was performed using Pearson’s chi-square test (*n* ≥ 5) or Fisher’s exact test (*n* < 5) with subsequent multiple comparisons using chi-square with the Bonferroni correction for categorical variables. One-way ANOVA with subsequent post hoc multiple comparisons was used for continuous variables with the Student–Newman–Keuls test.

Due to the small number of cells and a large number of apoptotic and necrotic cells in the VSTM1 overexpression group, only the apoptosis and necrosis of VSTM1 overexpressing cells without treatment with ox-LDL were measured. Compared with the control group (9.62 ± 1.98) and NC group (10.18 ± 2.02), the proportion of apoptotic and necrotic cells after infection with VSTM1 overexpression virus was obviously increased (36.95 ± 2.59) compared with the NC group (*n* = 6, *p* ＜ 0.0001; Figure 4a and b). Compared with the control group (30.18 ± 3.45) and NC group (31.07 ± 3.71), the apoptosis and necrosis of sh-VSTM1 U937 cells after treatment with ox-LDL were significantly reduced (6.41 ± 2.54; *n* = 6, *p* < 0.0001), suggesting that depletion of VSTM1 could significantly ameliorate the apoptosis and necrosis of U937 cells (Figure 4c–f).

#### 3.3.2 Effects of VSTM1 on the proliferation of monocytes/macrophages stimulated by ox-LDL

##### 3.3.2.1 Depletion of VSTM1 promotes proliferation of monocytes/macrophages

The relative absorbance ratio of the three groups of U937 cells (CCK-8 absorbance measured at 24 and 48 h/0 h) showed that the growth rate of the sh-VSTM1 group was higher compared with the control group and NC group (compared with the U937 group, *n* = 6, *p* = 0.0412; compared with the U937 NC group, *n* = 6, *p* = 0.0189 at 24 h; compared with the U937 group, *n* = 6, *p* = 0.0117; and compared with the U937 NC group, *n* = 6, *p* = 0.0071 at 48 h; Figure 4g and h).

##### 3.3.2.2 Inhibitory effect of ox-LDL on cell growth

After depletion of VSTM1, the inhibitory effect of ox-LDL on cell proliferation was reduced in the sh-VSTM1 group (compared with the U937 group, *n* = 6, *p* = 0.0048; the U937 NC group, *n* = 6, *p* = 0.0043 at 24 h; the U937 group, *n* = 6, *p* = 0.0028; the U937 NC group, *n* = 6, *p* = 0.0079 at 48 h; the U937 group, *n* = 6, *p* = 0.0011; and the U937 NC group, *n* = 6, *p* = 0.0001 at 72 h; Figure 4i and j). These results suggested that depletion of VSTM1 could attenuate the inhibitory effect of ox-LDL on cell proliferation.

### 3.4 Suppressed expression of VSTM1 critically aggravates monocytic dysfunction by mediating the NF-κB pathway

**Figure 5:**
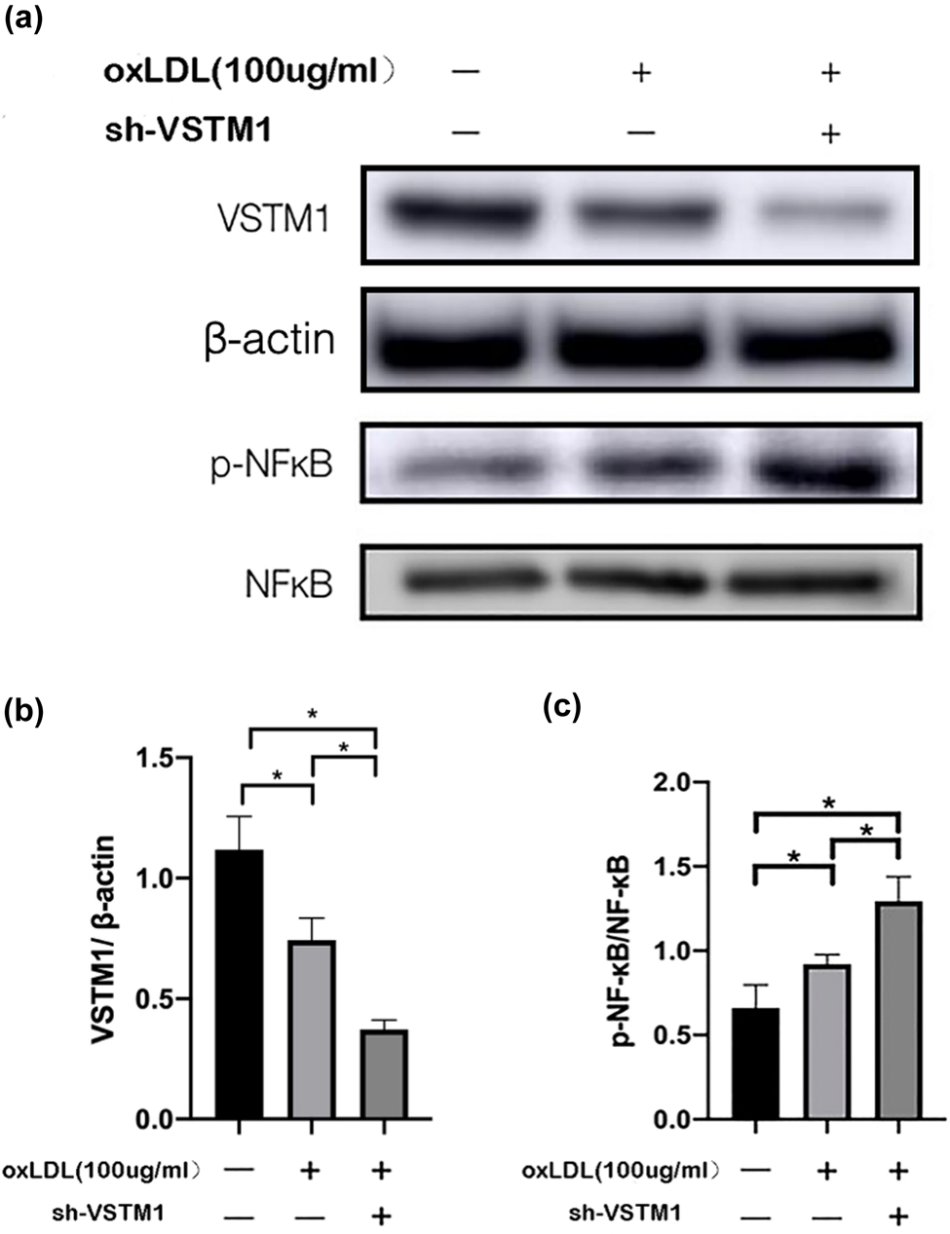
VSTM1 deficiency can induce the transcriptional activity of NF-κB (a–c). The expressions of VSTM1 and phospho-p65 in U937 cells were estimated by Western blotting analysis. U937 cells were treated with ox-LDL (100 µg/mL) and transfected with VSTM1 + ox-LDL (100 µg/mL), and the NC group was used as the controls (difference between groups, *p < 0.05, *n* = 5). Statistical analysis was performed using Pearson chi-square test (*n* ≥ 5) or Fisher’s exact test (*n* ＜ 5) with subsequent multiple comparisons using Chi-square with Bonferroni correction for categorical variables. One-way ANOVA with subsequent post hoc multiple comparisons was used for continuous variables with the Student–Newman–Keuls test.

The expression of VSTM1 at the protein level was suppressed when exposed ox-LDL (compared with the NC group, *n* = 6, *p* = 0.011), which was further inhibited in sh-VSTM1 U937 stable transfection cells after exposure to ox-LDL (compared with NC group, *n* = 6, *p* = 0.002). Additionally, under ox-LDL stimulation, the expression of phospho-NF-κB was increased (compared with the NC group, *n* = 6, *p* = 0.008). Furthermore, when the U937 cells were treated with ox-LDL for 24 h after sh-VSTM1 transfection, the expression of phospho-NF-κB was further increased compared with the NC and ox-LDL groups (compared with the NC group, *n* = 6, *p* < 0.0001, compared with the ox-LDL group, *n* = 6, *p* = 0.02). These results suggested that inflammation and other related stimuli could suppress the expression of VSTM1, leading to enhanced phosphorylation of NF-κB (Figure 5a–c).

## 4 Discussion

In this study, we explored the effects of VSTM1 on the biological function of monocytes/macrophages stimulated by ox-LDL. First, we used pLenti-VSTM1 and pLenti-VSTM1 shRNA viruses to transfect and identify effective monocytes/macrophages. The effects of VSTM1 on the proliferation, invasiveness, chemotaxis, and inflammation of human PBMCs/macrophages stimulated by ox-LDL were studied by a series of *in vitro* experiments. After VSTM1 was overexpressed in U937 cells, the growth rate of cells was significantly slowed down/stagnated, accompanied by a large number of apoptotic and necrotic cells. Therefore, only the depletion group was chosen for the subsequent study. We found that depletion of VSTM1 promoted the invasiveness and chemotaxis, as well as the inflammatory response of cells, and also ameliorated cell necrosis and apoptosis. Mechanistically, NF-κB was activated in VSTM1-depleted U937 cells.

It is well known that inflammation plays a critical role in the rupture of atherosclerotic plaque. AS is the inflammatory response of macrophages and monocytes to the “pathogenic lipoproteins” invading the arterial wall [13,14,15]. Macrophages promote the initiation and progression of AS injury. Therefore, the migration and invasiveness of macrophages are very important for the pathogenesis of AS. We found that the depletion of VSTM1 could significantly enhance the invasiveness and chemotaxis of macrophages stimulated by ox-LDL. Moreover, after exposure to ox-LDL, the secretion of IFN-α and MMP-9 was significantly increased. Ox-LDL increased the secretion of TNF-α and IL-8 in a concentration-dependent manner. These results suggested that depletion of VSTM1 could enhance inflammatory response stimulated by ox-LDL. As far as we know, no study has reported such findings worldwide.

The accumulation of ROS, also known as oxidative stress, is related to the occurrence of AS. AS is partially attributed to the imbalance of redox in the vascular system. In recent years, studies on chronic inflammatory diseases have shown that the selective induction of inflammation-related genes by intracellular oxidative stress is one of the common molecular mechanisms that causes AS [16]. Different oxide precursors can directly stimulate or sensitize vascular cells, leading to the production of living oxygen, which further promotes monocytes to infiltrate into the vascular wall and to release inflammatory molecules. The above-mentioned mechanisms form a positive feedback loop that continuously strengthens local inflammatory response, and also damages the physiological function of other cells. We found that the depletion of VSTM1 could increase the production of ROS, which was consistent with an earlier study that VSTM1 can reduce the production of ROS in neutrophils [17].

Apoptosis plays an important role in the pathogenesis of various cardiovascular diseases, especially in AS. It has been found that endothelial cells, smooth muscle cells, and macrophages all can undergo apoptosis and necrosis. In addition, apoptosis plays a leading role in the formation of atherosclerotic plaques [18,19]. In recent years, it has also been found that apoptosis is related to cell proliferation. Studies have found that cell apoptosis exists in plaques. The development of plaques depends on the relative balance between apoptosis and cell proliferation. Apoptosis in AS can affect vascular remodeling. Moreover, apoptosis in AS can directly affect the shape and structure of arteries and plays a very important role in plaque stability. In this study, we found that the depletion of VSTM1 could significantly reduce the incidence of cell necrosis and apoptosis, and attenuate the inhibitory effects of ox-LDL on cell proliferation.

NF-κB is an important nuclear transcription regulator, which is involved in a variety of physiological and pathological processes, and closely related to the expressions of various inflammatory genes. Many genes encoding metalloproteinases, proinflammatory factors, chemokines, and tissue factors are regulated by it. NF-κB is one of the transcription factors mainly involved in the expression and regulation of immune and inflammatory response molecules. It plays an important role in the regulation of immune response, inflammatory response, and cell growth [20]. Earlier studies have shown that NF-κB plays a fundamental role in the process of macrophage infiltration and atheromatous plaque formation [21]. The activation of NF-κB can enhance the transcription and production of a variety of inflammatory factors, which subsequently promote sustained inflammatory response [22]. The high expression of NF-κB in plaque indicates the aggravation of inflammation and the increased risk of plaque rupture [23]. In this study, we also found that the expression of MMP-9 was significantly increased after the depletion of VSTM1. MMP-9 is expressed in atherosclerotic tissue. Upon activation, MMP-9 may contribute to vascular remodeling and plaque rupture. Ox-LDL has been reported to upregulate MMP-9 and downregulate TIMP1 in monocyte-derived macrophages. This may contribute to matrix degradation in atherosclerotic plaques [24]. In this study, the expression of VSTM1 was inhibited by inflammation or related stimulation, and the transcriptional activity of NF-κB was increased. Taken together, we believed that VSTM1 might play a role in AS via modulating NF-κB signaling. These findings provided a new therapeutic target and angle for the treatment of coronary AS.

## 5 Conclusion

VSTM1 may play an important role in the activation of monocytes/macrophages, thus participating in the pathogenesis of AS. Dynamic monitoring of the VSTM1 expression on the monocyte surface may be a more effective way for the prevention and treatment of AS. The development of NF-κB inhibitors is expected to bring a breakthrough to the medical treatment of AS.

## 6 Limitations

In this study, we found that VSTM1 affects human monocytes via modulating NF-kB signaling. As monocytes play an important role in AS, our findings suggest a mechanism potentially involved in AS. However, this study was mainly composed of *in vitro* experiments and should be tested in blood samples of patients with AS. Therefore, our current findings still need to be verified in further *in vivo* experiments.
